# Pituitary function and the response to GH therapy in patients with Langerhans cell histiocytosis: analysis of the KIMS database

**DOI:** 10.1530/EJE-22-0160

**Published:** 2022-07-04

**Authors:** Philippe Touraine, Yempabou Sagna, Anders F Mattsson, Pia Burman, André P Van Beek, Martin Ove Carlsson, Ferah Aydin, Ulla Feldt-Rasmussen, Cecilia Camacho-Hübner

**Affiliations:** 1 Department of Endocrinology and Reproductive Medicine, Centre de Maladies Endocriniennes Rares de la Croissance et du Développement, Hôpital Universitaire Pitié Salpêtrière-Charles Foix, Sorbonne Université, Faculté de médecine, Paris Cedex 13, France; 2 Department of Internal Medicine, CHU Souro Sanou, Intitut Supérieur des Sciences de la Santé (IN.S.SA), Université Nazi Boni, Bobo-Dioulasso, Burkina Faso; 3 Pfizer Health AB, Sollentuna, Sweden; 4 Department of Endocrinology, Skåne University Hospital, University of Lund, Malmö, Sweden; 5 Department of Endocrinology, University Medical Center Groningen, University of Groningen, Groningen, Netherlands; 6 Department of Endocrinology and Metabolism, Rigshospitalet, and Institute of Clinical Sciences, Copenhagen University, Copenhagen, Denmark; 7 Pfizer Inc., New York, New York, USA

## Abstract

**Objective:**

To analyze the effectiveness and safety of growth hormone (GH) replacement treatment in adult patients with Langerhans cell histiocytosis (LCH) and GH deficiency (GHD) enrolled in KIMS (Pfizer International Metabolic Database).

**Patients and methods:**

Patients with LCH and GHD were studied at baseline and some of them after 1 year of GH treatment. The effectiveness of GH is presented as change after 1 year of treatment (mean, 95% CI). The LCH population was compared to two other groups of patients enrolled in KIMS, granulomatous and lymphocytic hypophysitis.

**Results:**

At baseline, 81 adults with LCH (27 with childhood onset, 56% females), mean age at GHD onset of 29 (15) years were studied. Diabetes insipidus was diagnosed in 86% of patients. Analysis of 1 year of GH treatment was possible in 37 patients. One-year cross-sectional values for the GH dose were 0.39 (s.d.± 0.21) mg and −0.5 (−1.2 to 0.2) for insulin-like growth factor-1 s.d. Total cholesterol decreased 0.9 (−1.5 to −0.3 (mmol/L); *P* < 0.05); AGHDA-QoL-score (*n* = 20) was improved by 2.8 points (−5.6 to 0.0; *P* < 0.05), while mean BMI increased 0.6 ± 3 kg/m^2^ (95% CI: −0.2 to 1.4). All these effects did not differ from the two other groups after adjusting for age, gender, and baseline values. In 20 of 77 patients included in the safety analysis, 36 serious adverse events were reported during 435 patient-years (82.8/1000); no new safety signals were reported.

**Conclusion:**

After 1 year of GH treatment in patients with LCH, metabolic variables and quality of life improved, with no new safety signals.

## Introduction

Langerhans cell histiocytosis (LCH) is a rare infiltrative myeloid neoplastic disease with variable clinical courses (1). Several small, retrospective studies describe endocrine manifestations associated with LCH but there are few published data in adult patients (2, 3, 4). Diabetes insipidus (DI) is the most frequent endocrine manifestation, and growth hormone (GH) deficiency (GHD) is the most frequent anterior pituitary dysfunction (2, 3, 4).

In adult patients, GHD leads to abdominal fat accumulation, decreased muscle mass, dyslipidemia, and a decline in wellbeing (5). GH replacement may improve quality of life (QoL), increase bone density, normalize body composition (lean and fat mass), and reduce total and LDL cholesterol without causing serious adverse effects (5). The administration of GH in children with LCH was found not to be associated with any alterations in disease activity and/or recurrences (6), but no corresponding published data exist in adults. Finally, there are no recommendations regarding the use of GH replacement in LCH adults (7).

In this context, we aimed to evaluate whether GH replacement was safe in LCH patients with GHD and whether GH replacement response (measured by serum insulin-like growth factor-1 (IGF-1) concentration, waist circumference, lipids, glucose, and QoL in this group differed from patients with two other infiltrative diseases, granulomatous and lymphocytic hypophysitis.

## Patients and methods

### Study groups

At baseline, 317 patients with GHD were enrolled in the study (Table 1). Among them, 226 were true or semi-naive patients for GH replacement and 91 were non-naive.

**Table 1:** Background. Dichotomous variables of all patients (*n* = 317).

	LCH	GLH	LYH	*P*-het*
Number of patients (%)	81	65	171	–
Females (%)	56	49	73	0.001
Pituitary deficiencies				
GHD severity	56	55	63	0.45
ADH deficiency	86	59	32	<0.0001
LH/FSH deficiency	67	89	75	0.007
TSH deficiency	61	84	83	0.0003
ACTH deficiency	48	81	78	<0.0001
Age at pituitary deficiency diagnosis	21 ± 16	33 ± 12	42 ± 13	<0.0001
Age at GHD diagnosis	29 ± 16	39 ± 13	44 ± 13	<0.0001
Age at KIMS entry	35 ± 13	42 ± 13	45 ± 12	<0.0001
Number of additional deficiencies	2.6 ± 1.2	3.1 ± 1.1	2.6 ± 1.1	0.005
Concomitant medications, *n* (%)				
Anti-lipid drugs	9 (8.6)	2 (1.5)	7 (7)	0.15
ACTH substitution therapy	28 (96.5)	46 (97.9)	101 (98.1)	0.80
ADH substitution therapy	51 (92.7)	29 (93.6)	40 (100)	0.23
LH/FSH substitution therapy	36 (87.8)	41 (87.2)	74 (79.6)	0.40
TSH substitution therapy	38 (100)	46 (97.9)	107 (96.4)	0.83

*P-value heterogeneity between group means (alternative hypothesis: at least one group mean is different from the others).

ACTH, adenocorticotropin hormone, ADH, anti-diuretic hormone, GH, growth hormone, GHD, growth hormone deficiency; GLH, granulomatous hypophysitis; LCH, Langerhans cell histiocytosis; LYH, lymphocytic hypophysitis; LH/FSH, luteinizing hormone/follicle-stimulating hormone; TSH, thyroid-stimulating hormone.

### LCH patient group

#### Baseline study

Eighty-one patients (excluding one case with missing data on birth date and gender) with a medical history of LCH and confirmed GHD were enrolled in the KIMS database (Pfizer International Metabolic Database, the largest registry and has been collecting information about adult patients with GHD both who receive and do not receive GH replacement therapy (8)). There was no previous cranial radiotherapy reported in the past medical history of the CRF. Patients were selected for treatment with GH according to real-life practice, of which 56% had confirmed severe GHD based on a GH peak <3 µg/L by an insulin tolerance or a glucagon test, on a GH peak <0.4 µg/L by an arginine test, or below the proposed BMI-related cut-off levels by a GHRH-arginine test (BMI <25; peak GH <11.5 µg/L; BMI 25–30 peak GH <8 µg/L; BMI >30 peak GH <4.2 µg/L) (5). Insulin-like growth factor-1 s.d. (IGF-I s.d.) <−2 was accepted as a criterion of GHD in case of three or more additional pituitary hormone deficiencies. All of these severe GHD cases had childhood onset, 95% had multiple pituitary deficiencies and 2% were isolated.

In non-severe GHD patients with childhood onset, 94% had multiple pituitary deficiency and 8% were isolated.

Gender, age at baseline, at the onset of GHD and pituitary disease, GH peak (maximum concentrations of serum GH), other pituitary hormone deficiencies, anti-diuretic hormone (ADH) deficiency, luteinizing hormone/follicle-stimulating hormone (LH/FSH) deficiency, thyroid-stimulating hormone (TSH) deficiency, and adrenocorticotropin hormone (ACTH) deficiency, were collected. Analyses were performed according to GH naivety (non-naive vs semi- or true-naive at KIMS baseline and only semi- and true-naive patients for 1-year effectiveness).

#### Effectiveness and safety study

Among the 81 patients enrolled in the database, the following were excluded for the effectiveness study: patients with less than 12 weeks of GH treatment or with delayed treatment start (*n* = 10), non-naive patients (e.g. treated with GH previously and/or during the 6-month period preceding KIMS start (*n* = 24), and patients for whom data after the 1-year visit were not available (*n* = 10). The final size of the effectiveness group was 37 patients.

For the safety study, we included all patients enrolled in KIMS with a GHD diagnosis of histiocytosis with at least one injection of GH and excluded four reference patients, four patients with LCH only in medical history (not related to GHD), and one with missing data of birth date and gender. The final size of the safety group at 1-year follow-up was 77 patients, and the patient-years of follow-up were 435 (median 4.1, range 0–16 years).

The following variables were collected at 1 year of GH treatment: body weight, serum IGF-I levels, IGF-I IGF-I s.d., total cholesterol, LDL cholesterol, HDL cholesterol, triglycerides, fasting blood glucose, QoL- assessment of GH deficiency in adults (QoL-AGHDA) scores.

All adverse events (AE) were reported, organized by MEDdra System Organ Class and preferred term.

### Comparison groups

For comparison, two groups of patients with GHD were used: granulomatous and lymphocytic hypophysitis. Corresponding criteria as for the LCH study group were applied: 236 patients had baseline characteristics, 65 with granulomatous and 171 with lymphocytic hypophysitis; 116 patients were included in the 1-year effectiveness and 231 in the safety study. Comparisons between groups were adjusted for age and gender. The approximate number of patient-years of follow-up was 1209 (median 4.4; range 0–16.5 years).

### Statistical methods

Descriptive statistics were presented with means and s.d., or proportions, depending on the type of variable.

Crude tests were performed through *t*-tests, chi-square, or Fisher’s exact test.

The statistical analyses of numerical baseline variables and 1-year numerical delta variables were performed by covariance analyses for unbalanced designs (PROC GLM, SAS version 9.4). Baseline comparisons, using mean values, were adjusted, in general, for age at baseline and gender. One-year delta analyses were additionally adjusted for the corresponding baseline value. Tests and 95% CIs were Wald based. *P* < 0.05 was considered statistically significant.

AE occurrence was presented as frequencies and incidence rates per 1000 patient-years. Patient-years were calculated from KIMS entry, or from the start of GH replacement if this occurred after KIMS entry, until the last visit, or date of death.

## Results

### Baseline characteristics

The mean age of the 81 patients with LCH (27 with childhood onset) was 21 years at diagnosis of the first pituitary deficiency and 29 years at diagnosis of GHD. Women represented 56% of the LCH group, 49% of the granulomatous group, and 73% of the lymphocytic hypophysitis group (*P*-value heterogeneity between group means: 0.001). Baseline characteristics of all patients are summarized in Table 1.

There were no significant interactions between GH naivety and study groups (Table 1) except for age at diagnosis of GHD, since non naïve patients were on average 4 years younger (*P* = 0.04).

Data on confirmation of severe GHD were present in 56% of the LCH patients, 55% of the granulomatous patients, and in 63% of the lymphocytic hypophysitis patients (*P*-value heterogeneity: 0.45). Additional pituitary deficiencies included ADH in 86%, LH/FSH in 67%, TSH in 61%, and ACTH in 48% of LCH patients (significantly higher for ADH and lower for the others – when comparing with patients with granulomatous and lymphocytic hypophysitis, Table 1).

The mean BMI was 27.9, 30.8, and 27.7 kg/m^2^, for LCH, granulomatous, and lymphocytic hypophysitis patients (*P* = 0.13), respectively.

The mean GH starting dose was 0.16 mg, 0.32 mg, and 0.21 mg, respectively, for the three groups (*P-*value heterogeneity = 0.04) (Table 2).

**Table 2:** Effectiveness endpoints. Baseline crude, unadjusted values at KIMS entry.

Baseline	LCH	GLH	LYH	*P*-het.^a^
Weight^b^, (kg)				
*n*	35	29	83	
Mean (s.d.)	75 (22)	95 (26)	82 (17)	0.0005
95% CI	69 to 82	88 to 103	77 to 86	–
BMI				
*n*	34	27	83	
Mean (s.d.)	27.4 (7.4)	30.8 (7.7)	27.7 (6.3)	0.21
95% CI	25.0 to 29.7	27.8 to 33.0	27.7 to 30.5	–
Total cholesterol, (mmol/L)				
*n*	14	17	42	
Mean (s.d.)	5.7 (1.2)	5.7 (1.4)	6.0 (1.5)	0.71
95% CI	4.9 to 6.4	5.0 to 6.4	5.5 to 6.4	
LDL cholesterol, (mmol/L)				
*n*	14	17	39	
Mean (s.d.)	3.3 (0.9)	3.5 (1.1)	3.6 (1.2)	0.76
95% CI	2.7 to 3.9	2.9 to 4.0	3.2 to 3.9	
HDL cholesterol, (mmol/L)				
*n*	14	17	42	
Mean (s.d.)	1.4 (0.4)	1.1 (0.4)	1.5 (0.5)	0.02
95% CI	1.2 to 1.6	0.9 to 1.3	1.3 to 1.6	
Triglycerides, (mmol/L)				
*n*	14	17	41	
Mean (s.d.)	2.1 (1.3)	2.6 (1.1)	1.8 (1.0)	0.04
95% CI	1.5 to 2.6	2.0 to 3.1	1.4 to 2.1	
Fasting blood glucose, (mmol/L)				
*n*	21	14	42	
Mean (s.d.)	4.9 (0.5)	4.7 (0.6)	4.9 (1.1)	0.81
95% CI	4.5 to 5.3	4.2 to 5.2	4.6 to 5.2	
QoL (AGHDA items)				
*n*	24	22	62	
Mean (s.d.)	9.3 (5.2)	11.3 (7.2)	11.3 (7.8)	0.50
95% CI	6.4 to 12.2	8.2 to 14.3	9.5 to 13.1	
IGF-I s.d.				
*n*	15	17	42	
Mean (s.d.)	−2.6 (2.0)	−1.7 (1.2)	−1.9 (1.7)	0.25
95% CI	−3.5 to −1.8	−2.5 to −0.9	−2.4 to −1.4	
% with ≤ −2s.d.	7 (50)	7 (46.7)	18 (46.2)	>0.99
GH dose^b^ (mg/day)				
*n*	37	31	85	
Mean (s.d.)	0.16 (0.12)	0.32 (0.47)	0.21 (0.18)	0.02
95% CI	0.10 to 0.26	0.23 to 0.41	0.12 to 0.23	

^a^ *P*-het: *P*-value heterogeneity between group means (alternative hypothesis: at least one group mean is different from the others); ^b^Weight and dose effects remained significant after adjusted for age and gender.

AGHDA, assessment of GH deficiency in adults; GH, growth hormone; GLH, granulomatous hypophysitis; IGF-I, insulin-like growth factor-1; LCH, Langerhans cell histiocytosis; LYH, lymphocytic hypophysitis; QoL, quality of life.

The crude mean of total cholesterol and LDL cholesterol for the true-naive and semi-naive LCH patients were 5.7 ± 1.9 mmol/L and 3.3 ± 0.9 mmol/L, respectively, and did not significantly differ between the study groups. The mean HDL cholesterol and triglycerides did not differ between the groups.

About concomitant medications, for anti-lipid drugs, there were 9, 2, and 7% in LCH, granulomatous, and lymphocytic hypophysitis patients, *P* = 0.15. The proportion of patients on ACTH, ADH, LH/FSH, and/or TSH medications ranged from 80 to 98% in the three groups of patients with non-missing dosing records, *P* > =0.23.

### Effectiveness study

One-year cross-sectional crude mean GH dose was 0.39 ± 0.21 mg/day for LCH patients and was similar to the other groups (Table 3).

**Table 3:** Effectiveness endpoints in patients with at least one year of follow-up (discrepancies to *n*  = 37, 31, and 85 depend on missing values).

Endpoint	Mean at overall baseline	Crude (unadjusted) values			
		LCH	GLH	LYH	*P*-het^a^
∆Weight^b^, kg	83				
*n*		33	29	81	
Mean (s.d.)		1.4 (8.2)	1.4 (6.6)	0.8 (5.5)	0.86
95% CI		−0.9 to 3.6	−1.0 to 3.8	−0.6 to 2.2	–
∆BMI, (kg/m^2^)	28.9				
*n*		32	27	81	
Mean (s.d.)		0.6 (3.0)	0.3 (2.0)	0.3 (2.0)	0.77
95% CI		−0.2 to 1.4	−0.6 to 1.13	−0.2 to 0.8	–
∆Total cholesterol mmol/L	5.9				
*n*		11	12	33	
Mean (s.d.)		−0.9 (0.9)	−0.6 (0.7)	−0.3 (1.2)	0.30
95% CI		−1.5 to −0.3	−1.2 to 0.1	−0.7 to 0.0	
∆LDL, (mmol/L)	3.5				
*n*		11	11	31	
Mean (s.d.)		−0.6 (0.7)	−0.4 (0.7)	−0.2 (0.8)	0.40
95% CI		−1.0 to −0.1	−0.8 to 0.1	−0.5 to 0.1	
∆HDL, (mmol/L)	1.4				
*n*		11	12	33	
Mean (s.d.)		−0.1 (0.3)	0.0 (0.1)	0.0 (0.4)	0.49
95% CI		−0.3 to 0.1	−0.2 to 0.1	−0.1 to 0.1	
∆Triglycerides, (mmol/L)	2.0				
*n*		11	12	32	
Mean (s.d.)		−0.5 (0.8)	−0.4 (0.7)	0.2 (0.7)	0.02
95% CI		−0.9 to −0.03	−0.8 to 0.05	−0.1 to 0.4	
∆F. Blood glucose, (mmol/L)	4.9				
*n*		13	6	28	
Mean (s.d.)		−0.1 (0.5)	0.5 (0.4)	0.3 (0.8)	0.21
95% CI		−0.5 to 0.3	−0.1 to 1.0	0.0 to 0.5	
∆QoL-AGHDA items	10.9				
*n*		20	18	42	
Mean (s.d.)		−2.8 (5.3)	−5.1 (7.1)	−3.6 (6.3)	0.53
95% CI		−5.6 to 0.0	−8.0 to −2.1	−5.5 to −1.6	
∆IGF-I s.d.	−2.0				
*n*		14	15	39	
Mean (s.d.)		1.9 (2.3)	2.2 (1.4)	2.1 (1.5)	0.93
95% CI		1.0 to 2.8	1.3 to 3.0	1.6 to 2.6	
∆GH dose, (mg/day)	0.20				
*n*		37	31	85	
Mean (s.d.)		0.21 (0.25)	0.14 (0.48)	0.24 (0.27)	0.32
95% CI		0.10 to 0.31	0.03 to 0.25	0.17 to 0.31	
1 year cross-sectional values					
IGF-I s.d.S at 1 year visit					
*n*		14	15	39	
Mean (s.d.)		−0.5 (1.3)	0.3 (1.2)	0.1 (1.2)	0.13
95% CI		−1.1 to 0.1	−0.3 to 0.8	−0.2 to 0.5	
% with ≤ −2 s.d.		5 (35.7)	1 (6.7)	3 (7.9)	>0.03
GH dose^b^ mg/day at 1 year					
*n*		37	31	85	
Mean (s.d.)		0.39 (0.21)	0.46 (0.52)	0.41 (0.27)	0.63
95% CI		0.28 to 0.49	0.35 to 0.58	0.34 to 0.48	

^a^ *P*-het: *P*-value heterogeneity between group means (alternative hypothesis: at least one group mean is different from the others). ^b^No effects remained significant after adjusted for age and gender. ∆: First-year delta (change).

∆, delta, AGHDA, assessment of GH deficiency in adults; GH, growth hormone; GLH, granulomatous hypophysitis; IGF-I s.d., insulin-like growth factor-1 s.d.; LCH, Langerhans cell histiocytosis; LYH, lymphocytic hypophysitis; QoL, quality of life.

The crude first-year delta of IGF-1 s.d. of LCH patients (Table 3) was 1.9 ± 2.3 and did not differ from the two other groups (Tables 2 and 3).

First-year delta BMI mean was 0.6 ± 3.0 kg/m^2^ (95% CI: −0.2 to 1.4) for LCH patients and did not differ for granulomatous and lymphocytic hypophysitis patients (both groups 0.3 ± 2.0 kg/m^2^, *P-*value for heterogeneity = 0.77). Based on few non-missing observations, there was an increase in lean tissue and a decrease in fat tissue in all the three subgroups (Table 3, no tests performed).

The first-year changes in total cholesterol, LDL cholesterol, triglycerides, and fasting glucose in the LCH group were −0.9 ± 0.9 mmol/L, −0.6 ± 0.7 mmol/L, −0.1 ± 0.3 mmol/L, −0.5 ± 0.8, and −0.1 ± 0.5 mmol/L, respectively. The changes were not different from the effects in the other two groups (Table 3).

The first-year mean delta QoL-AGHDA score of LCH patients was −2.8 ± 5.3 (−5.6 to 0.0; *P* < 0.05, reduction indicating improved QoL) and did not differ from the other groups (Table 3).

Effectiveness variables and their first-year deltas are summarized in Tables 2 and 3.

### Safety study

During 435 patient-years, 36 serious adverse events (SAEs) were reported by the investigators in 18 patients (82.8/1000 patient-years). Twenty-two of these 36 SAEs were associated with hospitalization (13 out of 20 patients), five reports indicated no hospitalization, and in nine, information on hospitalization was missing.

Twenty-seven SAEs were identified, and three deaths were reported (one from respiratory failure, one from alcohol abuse, and one from meningioma).

The most common SAEs were infections and infestations, psychiatric disorders, benign and malignant neoplasms, and unspecified and nervous system disorders (Table 4). No new safety signal was found.

**Table 4:** Incidence of serious adverse events for all patients with Langerhans cell histiocytosis on growth hormone treatment.

System organ class/ low-level term	Age (years) at		Causality to GH therapy^a^	Outcome
	GH start	SAE		
Gastrointestinal disorders (*n* = 1)				
Vomiting- After Intragastric balloon	51.1	52.7	No	Recovered
General disorders and administration site conditions (*n* = 2)				
Pyrexia	36.4	46.6	No	Recovered
Left leg thrombosis	33.6	36	No	Not recovered
Infections and infestations (*n* = 4)				
Ear infection	47.9	53.0	No	Recovered
Gastroenteritis	29.3	30.7	No	Recovered
Pneumonia	29.3	34.6	No	Recovered
Gastroenteritis	36.4	45.9	No	Recovered
Injury, poisoning, and procedural complications (*n* = 4)				
Road traffic accident	17.9	21.0	No	Recovered
Thoracic vertebral fracture	55.0	62.7	No	Recovered
Collapse after local anesthesia for dental procedure	68,6	69,9	No	Recovered
Joint injury	36.3	42.1	No	Not recovered
Metabolism and nutrition disorders (*n* = 2)				
Hyponatremia	36.3	42.1	No	Recovered
Hyponatremia	36.4	46.6	No	Recovered
Neoplasms benign, malignant, and unspecified (including cysts and polyps) (*n* = 3)				
Pituitary tumor recurrent	41.4	45.3	No	Recovered
Bone sarcoma – treated for primary condition with radiotherapy 10 years prior to SAE	38.1	44.6	UNK	UNK, GH therapy withdrawn
Meningioma				
Retroperitoneal bleeding. Patient not on GH therapy for 8 years prior to SAE	38.1	52.7	No	Death
Nervous system disorders (*n* = 3)				
Cerebral infarction	31.8	32.8	No	Recovered with Sequelae
CNS lesion	26.0	30.3	No	Recovered
Neurological symptom	36.4	45.9	No	Recovered
Pregnancy, puerperium and perinatal conditions (*n* = 1)				
Ectopic pregnancy	21.7	22.4	No	Recovered, GH treatment temporary withdrawal
Psychiatric disorders (*n* = 3)				
Alcohol abuse	55.0	67.0	Yes	Death
Depression	28.9	35	No	UNK
Anxiety crisis	21.8	22.8	No	Recovered
Respiratory, thoracic, and mediastinal disorders (*n* = 1)				
Respiratory failure	37.7	38.6	No	Death
Surgical and medical procedures (*n* = 4)				
Laparoscopy cholecystectomy	63.2	63.8	No	Recovered
Nasal septal operation	32.9	35.3	No	Recovered
Craniotomy	41.4	45.3	No	Recovered
Cyst removal	41.4	45.3	No	Recovered

^a^As reported by the investigator; some patients had more than one SAE as reported by the investigator.

GH, growth hormone; SAE, serious adverse event; UNK, unknown.

One patient had ACTH deficiency and two others had one more pituitary deficiency as additional pituitary dysfunction after 1 year of GH treatment.

For granulomatous hypophysitis, 35 SAEs have been reported in 23 patients during 352 patients-years (99.4/1000 patient-years). The most common SAEs were ‘infections and infestations’ and ‘general disorders and administration site conditions’. Twenty-six out of 35 SAEs required hospitalization (16 out of 23 patients) and 4 reported deaths.

Eighty-three SAEs have been reported in 46 lymphocytic hypophysitis patients for 857 patient-years (96.8/1000 patient-years). The most common SAE was nervous system disorders. Sixty-five out of 83 SAEs required hospitalization (38 out of 46 patients) and 7 reported deaths.

## Discussion

As reported in patients with granulomatous and lymphocytic hypophysitis, adult patients with LCH have multiple pituitary hormone deficiencies and ADH deficiency is often the first endocrine manifestation (4, 9, 10). GHD can occur in more than half of patients and is usually the most common anterior pituitary deficiency, followed by gonadotropin and TSH deficiencies (4, 7), Yet, there is limited information on the outcome of GH replacement therapy in this rare group of patients.

We assessed 1-year effectiveness and safety of GH replacement in adult patients with LCH in comparison with those with granulomatous and lymphocytic hypophysitis. The small number of the LCH patients included is a limitation of the study. Also, there were very few observations for a number of variables. Moreover, data confirming severe GHD were not present in about half of the cases, just over half of patients had confirmed severe GHD. Nevertheless, this study could provide valuable data on GH treatment for patients with this rare disease.

In the present study, the first-year mean GH dose was similar among the three groups of patients. Moreover, it was similar to the dose used in patients with GHD following treatment for pituitary adenomas (11, 12, 13). This dose was sufficient to achieve an increase in IGF-I s.d. compared to other studies (12, 13).

At baseline, the patients were often overweight or obese, a finding reported in other groups of GHD patients (13). In general, weight and BMI are unchanged by GH replacement therapy (12); however, when compared to age-matched healthy controls, GH treatment has shown beneficial effects over 4 years (14). Nevertheless, the use of GH in patients with GHD improves body composition with an increase in lean tissue and a decrease in total body fat (12, 15).

The typical effects of GH on lipid profile are a decrease in total and LDL cholesterol (16). In some studies, an increase or no change in HDL cholesterol has been reported (12, 13, 15), while most studies found no effect on triglycerides (12, 17). However, in the present study a reduction in triglycerides was demonstrated in all groups of patients.

A reduction in the mean fasting blood glucose was also found in the present study, albeit not significant. Data from the KIMS database previously demonstrated that high BMI and adverse body composition were associated with a higher prevalence of type 2 diabetes mellitus in adults with GHD and hypopituitarism (18). GH replacement seemed to have no positive effects on the glucose metabolism (17). However, according to a systematic review (11), there is moderate evidence for an increase in mean glucose levels after long-term GH therapy.

Studies on GH treatment of GHD have generally and consistently found improvement in QoL particularly within the first year of treatment (11, 12, 13, 15, 19, 20). The results of the present study were in keeping with these previous findings of reduced QoL-AGHDA score indicating an improved QoL in all three groups of patients.

GH replacement seemed safe (no new safety signals), but this small cohort was followed for a short time and there were no new safety concerns, without adversely affecting disease course. Infections and neoplasms were the most common SAEs found in this study, similar to previous studies (12, 13).

## Conclusion

Compared to granulomatous and lymphocytic hypophysitis patients, 1 year of GH replacement in adult patients with LCH and GHD was well tolerated and had positive effects on metabolic variables and QoL. If these findings can be corroborated by other (multicenter or registry) studies with more patients, long-term data and control group, GH replacement of GHD in LCH should be added to clinical practice guidelines.

## Declaration of interest

P T, P B, Av B, and U F R were members of the KIMS steering committee. A M, M C, and C C-H are, and N K and F A were fulltime employees of Pfizer Inc at the time of the study. Y S has nothing to disclose. U F R has received teaching honoraria from Novo Nordisk, IPSEN and Novartis. The research salary of UFR was sponsored by an unrestricted research grant from Kirsten and Freddy Johansen Foundation.

## Funding

This work did not receive any specific grant from any funding agency in the public, commercial, or not-for-profit sector.
